# Landscape Effects on the Presence, Abundance and Diversity of Mosquitoes in Mediterranean Wetlands

**DOI:** 10.1371/journal.pone.0128112

**Published:** 2015-06-18

**Authors:** David Roiz, Santiago Ruiz, Ramon Soriguer, Jordi Figuerola

**Affiliations:** 1 Estación Biológica de Doñana (CSIC), Isla de La Cartuja, Av. Américo Vespucio, s/n. 41092, Sevilla, Spain; 2 Diputación de Huelva, Área de Medio Ambiente, Servicio de Control de Mosquitos, Huelva, Spain; Metabiota, UNITED STATES

## Abstract

Environment determines the distribution of mosquito-borne diseases in that it influences the vector-host-pathogen transmission cycle, including vector distribution, abundance and diversity. In this study, we analyse the relationship between environmental variables estimated by remote sensing and the spatial distribution (presence, abundance and diversity) of seven mosquito species vectors of West Nile and other pathogens (Usutu, avian malaria and dirofilariasis) in the Doñana Natural Park, Spain. Traps were distributed over an area of 54,984 ha divided into six ecological units: marshland, sand dunes, scrubland, ricefields, crops and fishponds. We collected mosquitoes once a month from up to 112 locations using BG-Sentinel traps baited with BG-lure and CO_2_ during March-November 2010. Hydroperiod, NDVI and Inundation surface were estimated at several resolution scales (100, 250, 500, 1000 and 2000 metres) from corrected and normalized Landsat Images. We sampled 972,346 female mosquitoes, the most abundant species being *Culex theileri*, *Ochlerotatus caspius*, *Culex modestus*, *Culex perexiguus*, *Culex pipiens*, *Anopheles atroparvus* and *Ochlerotatus detritus*. Our results suggest that: (1) hydroperiod, inundation surface and NDVI are strongly related to the spatial distribution of mosquitoes; (2) the spatial scales used to measure these variables affected quantification of these relationships, the larger scale being more informative; (3) these relationships are species-specific; (4) hydroperiod is negatively related to mosquito presence and richness; (5) *Culex* abundance is positively related to hydroperiod; (6) NDVI is positively related to mosquito diversity, presence and abundance, except in the case of the two salt marsh species (*Oc*. *caspius* and *Oc*. *detritus*); and (7) inundation surfaces positively condition the abundance and richness of most species except the salt marsh mosquitoes. Remote sensing data provided reliable information for monitoring mosquito populations. Landscape significantly affected mosquito distribution and abundance, and as a result may alter disease risk. These results suggest that while environmental conditions affect the distribution and abundance of mosquitoes, other factors such as human modification of landscapes may give rise to significant changes in mosquito populations and consequently disease risk.

## Introduction

Mosquito-borne diseases are among the world’s major causes of illness and death, particularly in tropical and subtropical countries, although they are also (re)emerging in temperate areas. In recent years outbreaks of West Nile virus (WNV), Chikungunya, Dengue and Usutu virus (USUV) have occurred in the USA and/or Europe [1,2,3,4].

Each pathogen can only be successfully transmitted by a limited range of mosquito species and the distribution of these competent vector species determines the geographical distribution of the pathogen [5]. Different mosquito species may play different roles in the genesis of an epidemic, especially in the case of multi-vector/multi-host pathogens, such as WNV [6]. Some species may contribute to pathogen amplification during the enzootic cycle, while others may make a greater contribution to the epizootic cycle [7]. In addition, a recent theoretical model suggested that vector species richness may facilitate virus amplification [8]. Consequently, understanding the factors that condition vector distribution, richness and abundance is an important step in characterising the risk of transmission of vector borne diseases [9,10,11].

Mosquito spatial distribution is constrained by the distribution of aquatic environments that support the development of larvae, and by the factors that determine adult mosquito habitats (for example, vertebrate host distribution and vegetation). Remote Sensing is a powerful tool for characterising these habitats at both large and fine spatial scales in that it provides reliable, cheap and periodic estimates of many environmental variables. Images generated by Landsat thematic mapper (TM) with a spatial resolution of 30 m can be used to characterize larval habitats locally at a low spatial scale [12]. However, it is not only the distribution of water for mosquito habitats that is important but also vegetation type, and covertures [13,14]. NDVI (Normalized Difference Vegetation Index) is positively related to vegetation biomass and is also affected by vegetation composition, and NDVI is related to WNV human cases and *Culex* abundance [15,16,17]. Although the resolution of Landsat is greater than the size of some of the mosquito breeding habitats (such as small ponds or water deposits), most breeding sites in wetlands are captured by Landsat satellite images, which are less appropriate for urban environments. These data can also be used to calculate hydroperiod and inundation surfaces by comparing time series of satellite images [18]. NDVI estimates derived from Landsat Satellite images have been successfully used to predict mosquito populations [16,19, 20, 21], especially in non-urban environments. However, to the best of our knowledge, Landsat satellite-derived information on hydroperiod duration and inundation surfaces has not yet been used to analyse mosquito presence, abundance or diversity.

Wetlands have abundant resident and migratory bird and mosquito populations overlapping in space and time [20, 21] making them important ecosystems for the enzootic cycles of WNV and USUV transmission. Doñana is one of the largest wetlands in Europe with large areas of seasonal freshwater and brackish marshes as well as marshes under tidal influence. This system provides a unique opportunity to study the distribution of mosquito populations in relation to landscape composition. WNV is endemically circulating in birds and horses in this area [22, 23, 24], and horse and human disease cases have been recently reported in the neighbouring provinces [25]. Four *Culex* species in the area are potential WNV vectors: *Culex pipiens*, *Culex modestus*, *Culex perexiguus (univitattus)* and *Culex theileri* [7]. *Cx*. *perexiguus*, *Cx*. *modestus* and *Cx*. *pipiens* were the most important ornithophilic mosquito species for WNV enzootic circulation in Doñana, *Cx*. *perexiguus* being an important species for transmission to horses, while *Cx*. *pipiens* may play an important role in epizootic transmission to humans [7]. The screening of mosquitoes captured in Doñana for flavivirus revealed a new lineage of WNV in *Cx*. *pipiens*, WNV lineage 1 and Usutu virus in *Cx*. *perexiguus* [26, 27], a flavivirus of potential medical concern in *Ochlerotatus caspius*, and several insect flaviviruses (*Culex*FV) [28].

In this paper, we analyse the relationship between landscape and environmental variables estimated from satellite images and mosquito distribution, abundance and diversity.

## Material and Methods

### Study area

**Fig 1:**
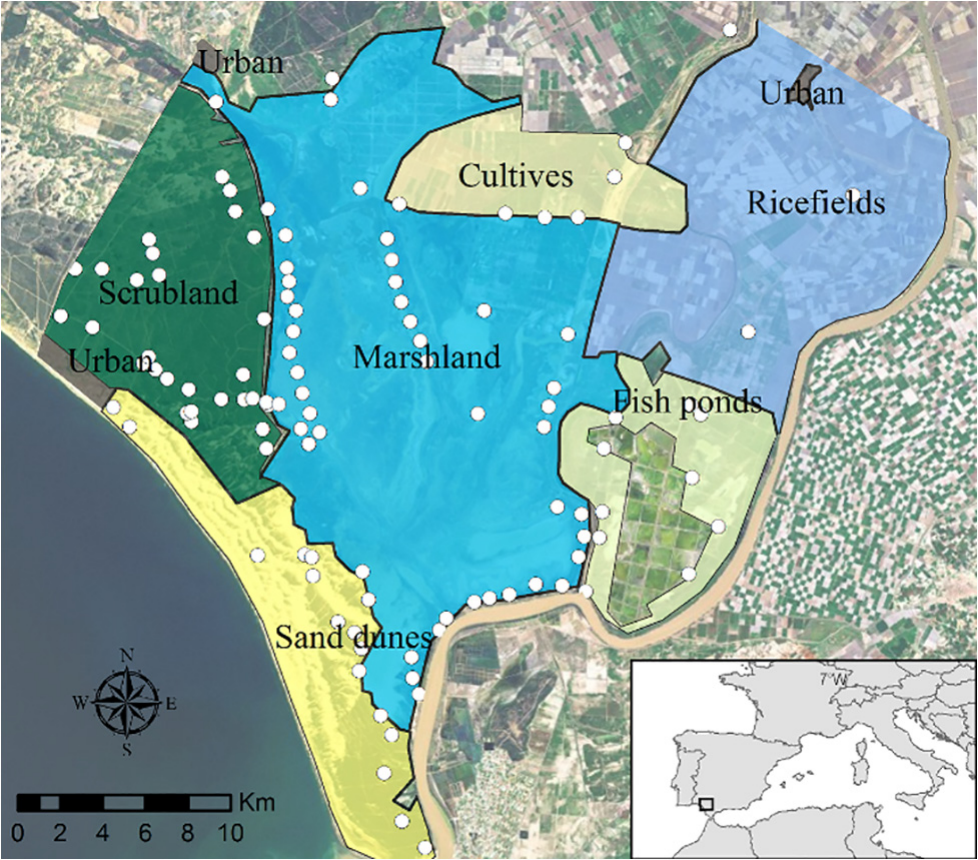
Map of the study area with an ortophotograph of the area. Ecological units are described in Material and Methods. Urban areas correspond to the villages situated at the border of the study area. White circles indicate the locations of the traps.

The regional Ministry of Agriculture, Fishing and Environment and Doñana Nature Area issue the permission for field work in the Doñana area (National Park and Natural Park). The Doñana Natural Space extends over more than 1,060 km^2^ and comprises two different areas: the Doñana National Park (54,251 ha) and the Doñana Natural Park (53,709 ha) that surrounds it. The National Park is strictly protected and has been declared a Biosphere Reserve, Ramsar Site and UNESCO World Heritage Site. A major site for migrating birds, it is considered one of the most important reserves and outstanding protected areas in Europe, attracting around 350,000 human visitors per year. The climate is Mediterranean sub-humid with rainy winters and dry summers. In 2009/2010 the hydrological cycle began in September and reached maximum inundation levels the following March. In late spring, evaporation becomes the most important factor in the water balance and the marshes dry up during the summer. The total sampling area covered 54,984 ha, divided into six ecological units or substrates (Fig 1).

1) Marshland (freshwater and salt marshes, 22,109 ha).The seasonal marsh floods when heavy rains arrive between October and March and is generally dry between July and October. Two different substrates were considered in the design: freshwater marshes (compose by *Scirpus maritimus*, *Scirpus litoralis*, *Typha latifolia and Phragmites australis*) and saltmarshes (with *Spartina densiflora*, *Salicornia ramossissima*, *Sarcocornia perennis*, *Artrhocnemum macrostachyum*), although they were analysed as asingle unit. Freshwater marshes are inundated by rainfall while saltmarshes depend on rainfall and tide regimens. 2) *Scrubland* (11,582 Ha). Forest and scrub over stabilized sands consisting of low woodland and interdunal valleys. 3) *Sand dunes* (9,113 ha). This area is a dune ecosystem 25 km in length which includes the shoreline. 4) *Fish ponds* (5,473 ha). Mainly brackish fish ponds with a semi-permanent flooding regime, and smaller areas of natural marshes. 5) *Crops* (5,207 ha). Dry-land crops surrounding the far northern end of the remaining natural marshes. 6) *Ricefields* (36,600 ha). Rice cultivation begins in May when the fields are flooded and ends around October when the rice is harvested.

### Sampling design

Adult mosquito distribution and abundance were determined using a stratified random sampling design, considered an efficient method for this purpose [29]. Each of the six ecological units was divided into 1x1 km squares and 20% of the quadrates of each unit were randomly selected. As some of these randomly selected quadrates (less than 2%) could not be easily reached walking or by car we exchanged them by others, with easier access, in the same ecological unit and chosen with the same randomization procedure. Each quadrate was located in the field using a PDA device with GPS and ArcPad sofware. BG-Sentinel trap baited with BG-lure and a container of dry ice (BG hereon) was placed in each of these quadrates, where possible in similar microhabitats (open areas near vegetation), and put into operation one night a month (from March to November 2010), for a total sampling effort of 716 trap nights. BG and CDC-CO^2^ traps have similar levels of efficacy for these mosquito species [30]. Trap number and distribution was adapted to the inundation regimes to omit areas inaccessible due to extensive flooding. A greater number of quadrates were sampled through the spring as the marsh dried until September when the maximum numbers of quadrates were sampled. During April, May and June, traps were transported by horse and placed on two transects across the flooded marshes. Due to the environmental homogeneity of the ricefields and the difficulty of maintaining the traps in these areas because of theft, fewer traps were located there. A total of 112 traps were sited, the numbers in each unit in proportion to their surface areas: 47 in marshland, 31 in scrubland, 15 in sand dunes, 10 in fish ponds, 6 in crops and 3 in ricefields.

### Mosquito identification

Traps were left in place for 24 hours, and then adult mosquitoes were collected, transported in dry ice to the laboratory and stored at -80°C. Insects were placed on a piece of white filter paper on a Petri plate over a chill table under a stereomicroscope for identification. Species was determined using taxonomic keys [31, 32]. Specimens belonging to the *univittatus* complex were assigned to the *Culex perexiguus* species on the basis of male genitalia, following Harbach (1999) [31, 32, 33]. When there were very large numbers of mosquitoes (over 500) we identified only 500 individuals while the rest of the sample was weighted and the proportion of identified individuals of each species extrapolated. Mosquito abundance was calculated as the mean of the number of females captured per trap night. All female abundance data was georeferenced and incorporated into a Geographic Information system (ArcGIS 10). We developed statistical models for the seven most important species: potential West Nile vectors *Culex perexiguus*, *Cx*. *modestus*, *Cx*. *pipiens* and *Cx*. *theileri* [7], the saltmarsh mosquitoes *Ochlerotatus caspius* and *Oc*. *detritus*, and the malaria vector *Anopheles atroparvus*.

### Remote sensing

Remote sensing variables were extracted from Landsat Images obtained from the USGS (U.S. Geological Survey) Geographic Information Centre (http://glovis.usgs.gov/). Twelve images were selected according to availability and cloud cover, spanning the full hydrologic year and the mosquito season (30 August 2009, 17 October 2009, 2 November 2009, 4 December 2009, 6 February 2010, 3 April 2010, 5 May 2010, 6 June 2010, 30 June 2010, 1 August 2010, 10 September 2010 and 5 November 2010). The Images were geometrically and radiometrically corrected and radiometrically normalized by the GIS and Remote Sensing Lab of the Doñana Biological Station (LAST-EBD) [18]. Hydroperiod, a variable that quantifies the number of days that each pixel (900 m^2^) remained flooded [34], was estimated from all the available images for the period 30 August 2009-September 2010 (Hydrological season). Inundation area was calculated as the number of pixels that were flooded inside each buffer. Areas unsuitable for mosquito larval development, such as salt ponds, the sea and the river, were removed from the analysis. Normalized Difference Vegetation Index (NDVI) was calculated from the images as a normalized ratio of the red and near-infrared bands. NDVI, which is commonly used to determine vegetation covertures, is a measure of the relationship between these two wavelength bands, from which photosynthetically active radiation can be calculated. Selection of a biologically appropriate buffer size has been shown to be important in identifying habitat predictors of species distribution [35]. Therefore, we constructed buffers of 100, 250, 500, 1000 and 2000 m in radius around each trap and calculated the following variables: 1) Annual hydroperiod 2) Annual NDVI 3) Monthly inundation surface and 5) Monthly NDVI. For these variables, the mean and standard deviation at the 2000 m. buffer were: 75.7±54.2 for annual hydroperiod; 0.16±0.19 for annual NDVI, 5.88±0.98 for monthly inundation surface and 0.07±0.06 for monthly NDVI. Using ARCGIS 10.1 software we extracted data for the various buffers using the Spatial Analyst tool Zonal Statistics as a table for raster files, and the Geoprocessing Intersect tool for vector files, and using a spherical variogram for the kriging method.

### Statistical analysis

Environmental variables may have a strong influence on mosquito populations not only at the time of adult capture but also, and mainly, at egg-laying and larval development which occur 1–4 weeks earlier [36]. For this reason, we examined the relationship between monthly mosquito data and environmental data from the current and previous months (Inundation surface and NDVI of the previous month). All the statistical analyses and figures were carried out in R version 2.14.2 (R Development Core Team 2005) using the mass, mgcv, lattice, pscl, ncf, rms, mumin and vgam packages.

#### Annual mosquito presence, abundance, diversity and richness

Our aim is to elucidate the relationships between environmental variables and several response variables: 1) Annual presence of each mosquito species (except *Cx*. *theileri*, present in all trapping locations), 2) Annual abundance of each mosquito species, and 3) Annual Shannon diversity index and Annual species richness. The independent variables included in these analyses were: a) mean annual hydroperiod, b) mean annual NDVI at five different buffers (100, 250, 500, 1000 and 2000 m.) and c) Landscape Unit. These relationships were analysed using: 1) a Generalized Linear Model (GLM) with logistic link and binomial distributed error [37] for annual mosquito presence, 2) a GLM with logarithm link and negative binomial distributed error for annual mosquito abundance, and 3) a GLM with identity link and normal distributed error for annual Shannon diversity index and for 4) mosquito species richness.

Due to the high co-linearity between some of the explanatory variables (i.e. the same variable calculated over different buffers or scales), models were fitted separately for each buffer and to each dependent variable: species presence, species abundance, Shannon Diversity Index and species richness. For each buffer, non-significant predictor variables were excluded stepwise from the saturated model using the ‘drop1’ command that drops each explanatory variable in turn and each time applies an analysis of deviance (Chi-squared distribution test). We included linear and quadratic terms and checked the VIFs (Variance Inflation Factors) [38], which were below 2 in all the models. We then selected the most informative buffer by AICc (corrected Akaike Information Criteria) using the ‘model.sel’ command (MuMin package). We validated the best models by checking for deviance homogeneity, normality, independence, influential observations, and overdispersion and spatial autocorrelation of the residuals. We calculated the explained deviance as: (Null deviance-Residual deviance)/ Null deviance).

#### Monthly mosquito presence, total abundance, diversity and richness

We fitted models to test the relationship between monthly presence of the seven commonest mosquito species and the explanatory variables: a) Inundation surface of the current month, b) Inundation surface of the previous month, c) NDVI of the current month, d) NDVI of the previous month, e) Month, and f) Landscape Unit. Generalized Linear Mixed Models (GLMM) with a binomial error distribution and binomial link were fitted including trap code as a random factor. GLMMs were used to control for pseudo-replication as traps were repeatedly sampled. Similar models were fitted using mosquito abundance and mosquito diversity (Shannon Index) as dependent variables. For these variables the GLMM models were fitted using a negative-binomial error distribution and logarithmic link. Separated models were constructed for each buffer of each dependent variable, non-significant predictor variables were excluded using the ‘drop1’ command, and selection of the most informative buffer was based on AICc. Model validation was performed as for annual data.

## Results

**Fig 2:**
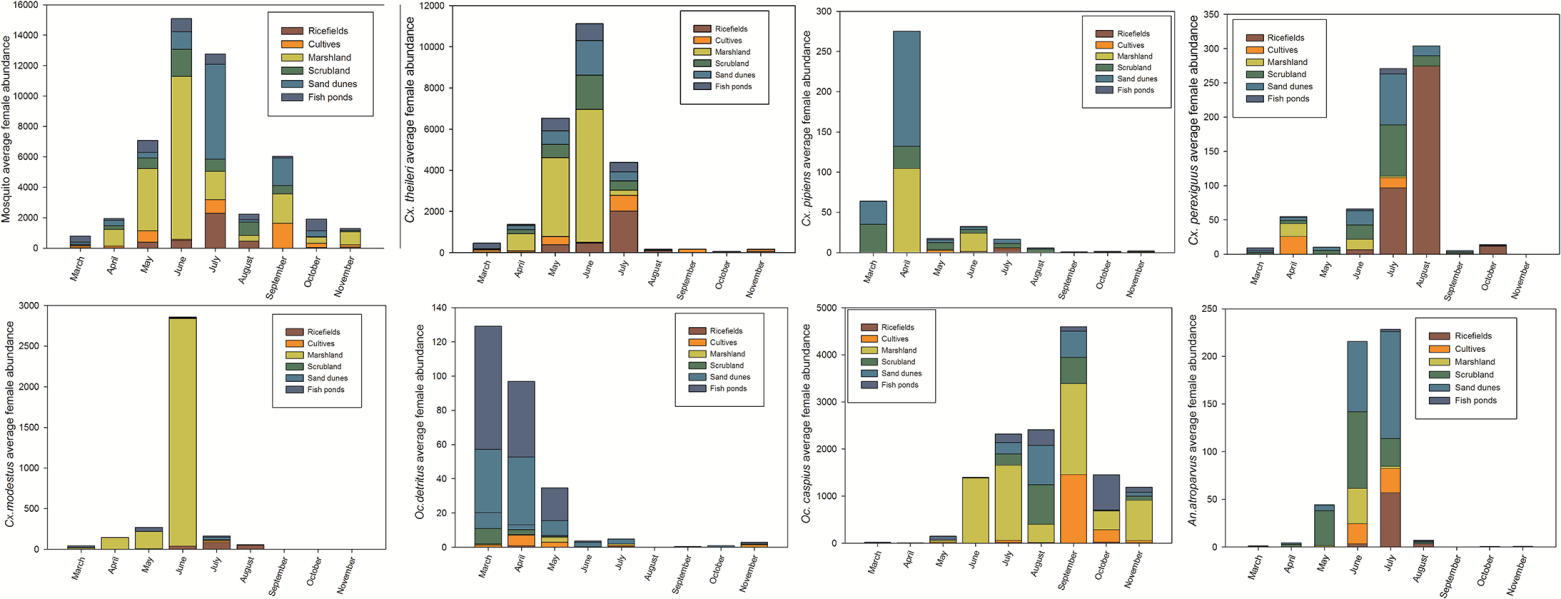
Monthly number of captures of the different mosquito species from March to November 2010 in the different ecological units studied. *Cx*. *theileri* was more abundant in marshland, but also in other landscape units. *Cx*. *perexiguus* was more abundant in ricefields, scrubland and other landscapes. *Cx*. *modestus* was more abundant in marshland and in June. *Cx*.*pipiens* was abundant in marshland, sand dunes and scrubland, *Oc*. *caspius* was abundant in all landscapes, while *Oc*. *detritus* was significantly more abundant in halophytic marshes of sand dunes and fish ponds.

**Fig 3:**
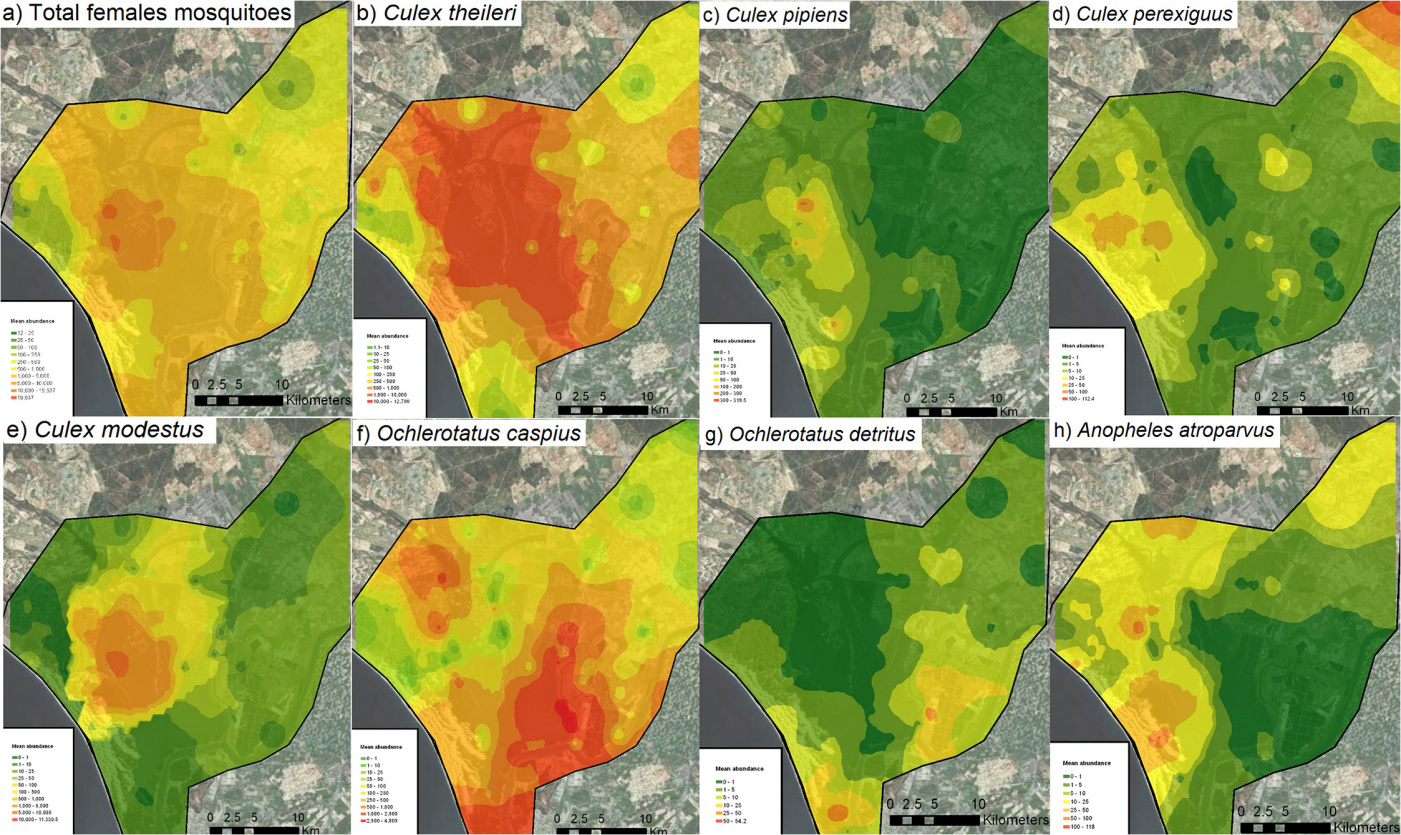
Spatial interpolation by kriging of annual mosquito abundances of: total female mosquitoes (a), *Cx*. *theileri* (b), *Cx*. *pipiens* (c), *Cx*. *perexiguus* (d), *Cx*. *modestus* (e), *Oc*. *caspius* (f), *Oc*. *detritus* (g), and *An*. *atroparvus* (h) with an ortophotograph of the area.

A total of 972,346 female mosquitoes were captured in 718 trap nights. The most abundant species, in descending order, were *Cx*. *theileri* (50.1%), *Oc*. *caspius* (37.4%), *Cx*. *modestus* (10.7%), *An*. *atroparvus* (0.9%), *Cx*. *perexiguus* (0.7%), *Cx*. *pipiens* (0.7%) and *Oc*. *detritus* (0.3%) (Table 1). *Culiseta annulata*, *Cs*. *subochrea*, *Cs*. *longiareolata*, *An*. *algeriensis* and *Uranotaenia unguiculata* accounted for 155 individuals and were therefore not analysed at species level. However, all the mosquito species were included in the estimation of mosquito diversity and richness. Average female mosquito abundance peaks in June-July with notable heterogeneity among the different landscape units, and spatial and temporal differences between individual mosquito species (Fig 2 and Fig 3). *Cx*. *theileri* is a “spring-season” mosquito species, its maximum abundance occurring during May, June and July, especially in marshland and to a lesser extent in Scrubland, sand dunes and other areas. *Cx*. *pipiens* is abundant in April in marshland and sand dunes; *Cx*. *perexiguus* is common during July and August in ricefields but also in July in scrubland and sand dunes. *Cx*. *modestus* is abundant in marshland during June. *Oc*. *detritus* is an early-season species, common from March to May in sand dunes and fishponds and near salt marshes, while *Oc*. *caspius* is abundant from June to November in all the areas, the adults dispersing from their larval breeding sites in salt marshes. *An*. *atroparvus* is common in June-July, especially in sand dunes and scrubland. Annual and monthly database are avalaible as supporting information (S1 Data and S2 Data).

**Table 1:** Number of female mosquitoes captured in each ecological unit.

Unit	*Culextheileri*	*Culex pipiens*	*Culex perexiguus*	*Culex modestus*	*Ochlerotatus caspius*	*Ochlerotatus detritus*	*Anopheles troparvus*	Total female mosquitoes
**Ricefields**	6199	19	797	399	166	3	132	7715
**Cultives**	6003	17	201	56	5824	61	142	12304
**Marshland**	316632	1973	836	101744	181755	111	1339	604390
**Scrubland**	96535	2219	4010	275	54210	283	4903	162435
**Sand dunes**	40886	2469	905	675	108531	1112	2914	157492
**Fishponds**	21360	27	148	893	13664	1349	32	37473
**Total general**	487615	6724	6897	104042	364149	2919	9462	972346

### Annual data analysis

**Fig 4:**
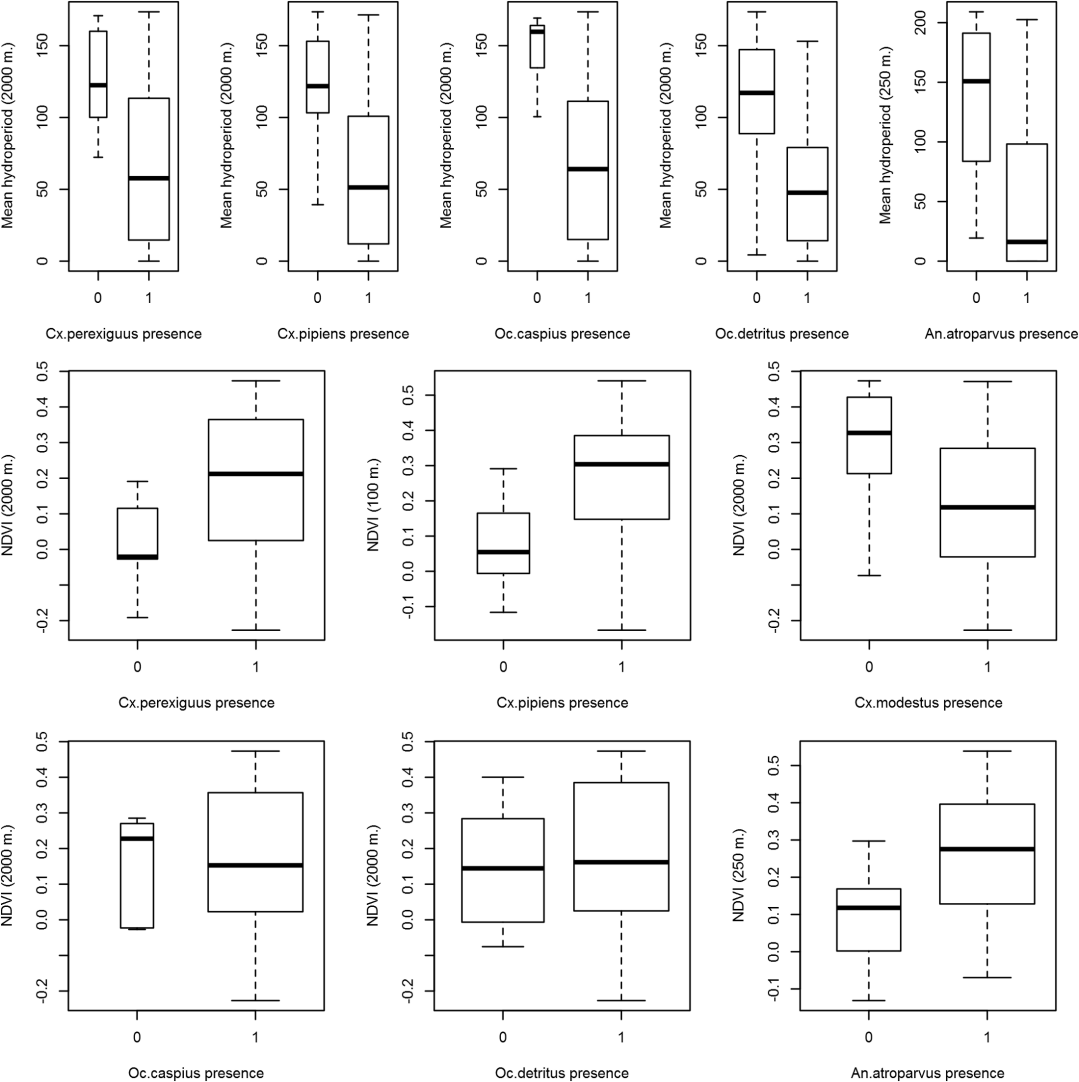
Results of the GLM binomial analysis of the influence of Hydroperiod and NDVI on annual mosquito presences in the five different buffers.

Hydroperiod was negatively related to the presence of all the species except *Cx*. *modestus* (Table 2; Fig 4). NDVI had a positive relationship with *Cx*. *perexiguus*, *Cx*. *pipiens* and *An*. *atroparvus* presence and a negative relationship with the presence of *Cx*. *modestus* and the two salt marsh species (*Oc*. *caspius and Oc*. *detritus*) (Table 2; Fig 4). Interestingly, 2000 m was the optimal buffer for most species, although for *An*. *atroparvus* and *Cx*.*pipiens* (250 m and 100 m. respectively) The models on presence successfully controlled for spatial autocorrelation and explained on average 40.4% of variance (Table 2).

**Table 2:** Results of the Generalized Linear Model (binomial) of annual presence for six mosquito species (*Cx*. *theileri* is not analysed) vs hydroperiod and NDVI estimated at five buffers of different size. NDVI^2^ is the quadratic effect of NDVI.

Dependent variable	Independent variable	Estimate	Std.error	Z value	p	Explained Deviance
***Culex perexiguus***	Intercept	3.001854	1.095491	2.740	0.00614	41.6%
	Hydroperiod (2000 m.)	-0.020864	0.008663	-2.408	0.01602	
	NDVI^2^ (2000 m.)	34.165384	15.016142	2.275	0.02289	
***Culex pipiens***	Intercept	2.03043	0.82707	2.455	0.01409	45.2%
	Hydroperiod (2000 m.)	-0.01907	0.00669	-2.850	0.00437	
	NDVI^2^ (100 m.)	24.89510	8.71846	2.855	0.00430	
***Culex modestus***	Intercept	2.7754	0.5385	5.154	<0.001	21.9%
	NDVI^2^ (2000 m.)	-5.6077	1.7095	-3.280	0.0010	
***Anopheles atroparvus***	Intercept	1.68936	0.61376	2.752	0.0059	36.4%
	Hydroperiod (250 m.)	-0.01684	0.00409	-4.117	<0.0001	
	NDVI (250 m.)	4.15721	1.83953	2.26	0.0238	
***Ochlerotatus caspius***	Intercept	10.26935	2.40986	4.261	<0.0001	55.3%
	Hydroperiod (2000 m.)	-0.06248	0.01611	-3.879	0.0001	
	NDVI (2000m.)	-6.76464	3.13052	-2.161	0.0307	
***Ochlerotatus detritus***	Intercept	3.941303	0.896773	4.395	<0.0001	42.1%
	Hydroperiod (2000 m.)	-0.034884	0.007483	-4.662	<0.0001	
	NDVI (2000m.)	-5.41918	1.914628	-2.83	0.0046	

**Fig 5:**
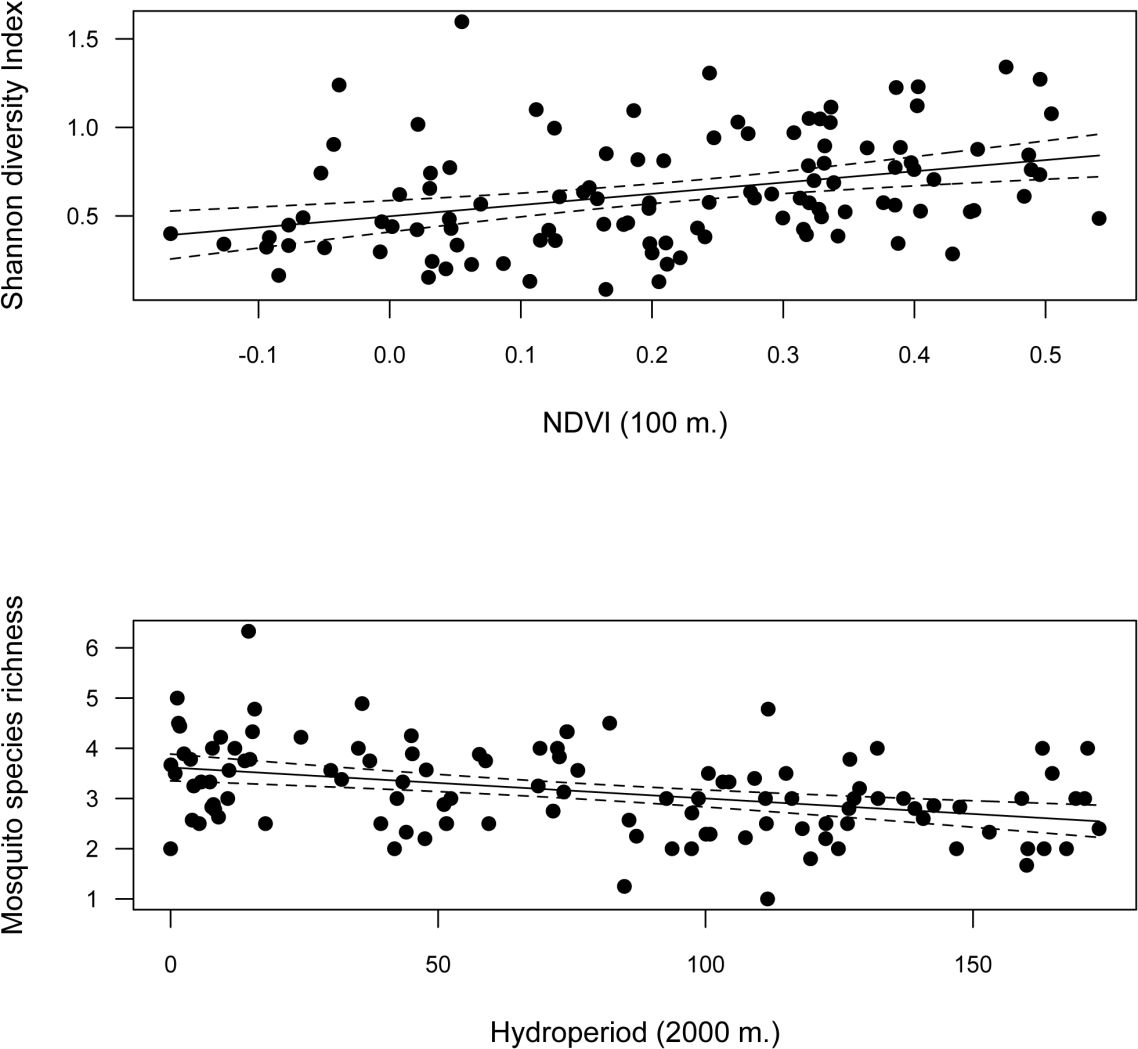
Relationships among annual hydroperiod, NDVI and Shannon diversity index and mean annual mosquito species richness.

**Fig 6:**
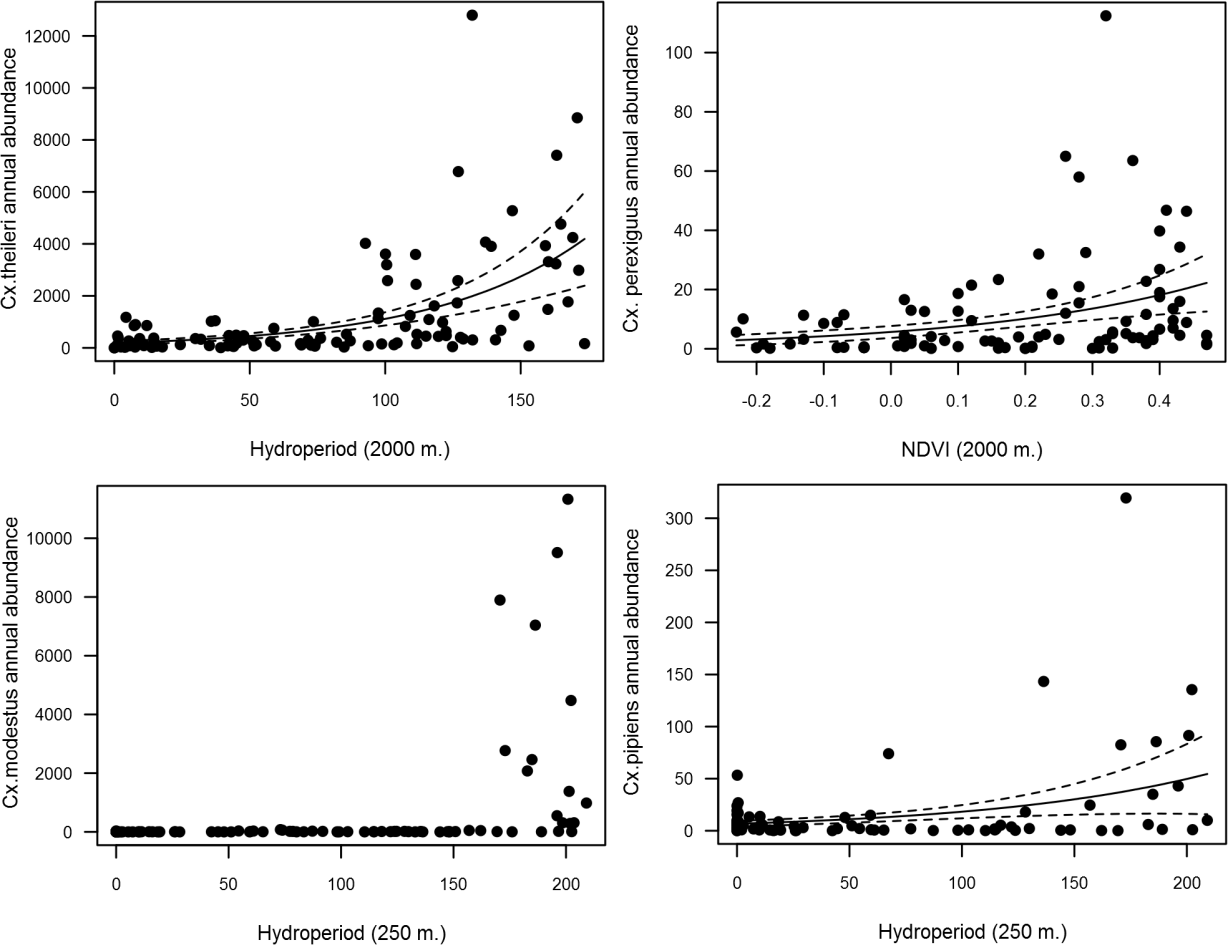
Relationships among annual hydroperiod, NDVI, *Cx*.*theileri*, *Cx*. *pipiens*, *Cx*.*modestus*, *Cx*.*perexiguus* and *Oc*.*caspius* annual abundances.

Annual NDVI was positively related to the annual Shannon diversity Index (Fig 5; Table 3). Interestingly, the model with the lowest AICc included the predictor variables calculated for the smaller buffer (100 m) while the AICc increased when data from larger buffers were included. On the other hand, annual hydroperiod (2000 m) was negatively related to mosquito richness (Fig 5, Table 3). *Cx*. *theileri*, *Cx*. *modestus* and *Cx*. *pipiens* annual abundances had a positive relationship with hydroperiod (Table 3, Fig 6), while *Cx*. *modestus* was more abundant in areas with long hydroperiods with threshold values of 150–250 days (Fig 6). *Cx*. *perexiguus* annual abundance had a positive relationship with NDVI (Fig 6), whereas *Oc*. *caspius* had a negative relationship with NDVI (Table 3). Final models on abundance explained on average 40.2% of variance. Abundances of each mosquito species varied across the different Landscape units (see Fig 2).

**Table 3:** Results of the Generalized Linear Model (normal) of diversity and mosquito species richness in relation to Hydroperiod and NDVI estimated at five buffers of different size; and results of the Generalized Linear Model (negative binomial) of annual female abundance for five mosquito species in relation to Hydroperiod and NDVI.

Dependent variable	Independent variable	Estimate	Std.error	Z value	p	Explained Deviance
**Shannon diversity index**	Intercept	0.49805	0.04446	11.203		22.5%
	NDVI (100 m.)	0.63493	0.16223	3.914	0.000159	
**Species Richness**	Intercept	3.6184	0.133408	27.124	<0.0001	39.1%
	Hydroperiod (2000 m.)	-0.006174	0.001435	-4.301	<0.0001	
***Culex theileri***	Intercept	5.219867	0.179935	29.010	<0.0001	47.6%
	Hydroperiod (2000 m.)	0.017979	0.001929	9.321	<0.0001	
***Culex modestus***	Intercept	0.862030	0.272589	3.162	0.0015	63.4%
	Hydroperiod (250 m.)	0.033984	0.002371	14.333	<0.0001	
***Culex perexiguus***	Intercept	1.7362	0.1778	9.766	<0.0001	20.9%
	NDVI (2000m.)	2.9063	0.6491	4.477	<0.0001	
***Culex pipiens***	Intercept	1.904844	0.206294	9.234	<0.0001	43.6%
	Hydroperiod (250 m.)	0.010010	0.002186	4.578		
***Ochlerotatus caspius***	Intercept	6.5793	0.1900	34.634	<0.0001	25.6%
	NDVI (2000m.)	-1.8079	0.7406	-2.441	0.0146	

### Monthly data analysis

**Fig 7:**
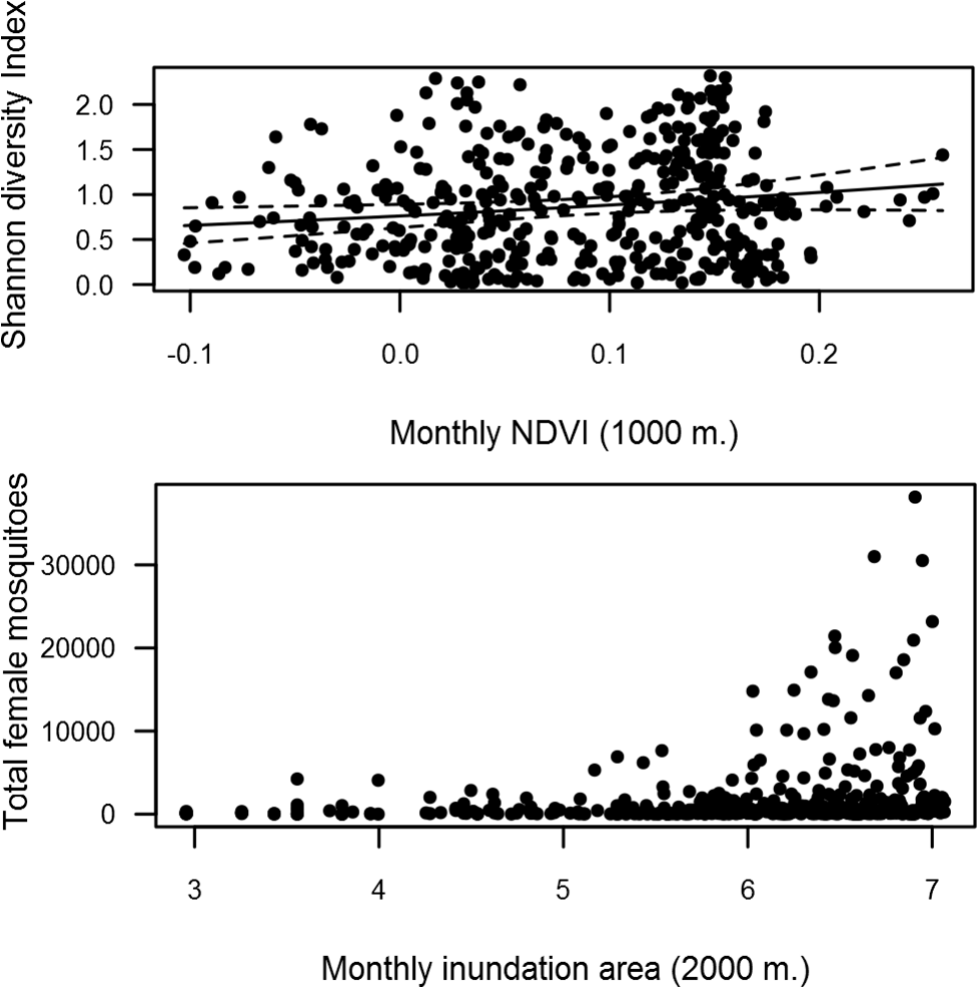
Relationships between Shannon diversity Index and monthly NDVI, and between monthly mosquito abundance and inundation area.

Inundation area was positively related to the monthly presence of *Cx*. *modestus*, *Cx*. *theileri*, *An*. *atroparvus* and *Oc*. *detritus*, and negatively related to the presence of *Cx*. *perexiguus* and *Oc*. *caspius*. NDVI had a positive relationship with the monthly presence of all species except *Oc*. *caspius* and *Cx*. *modestus* and the final models explained on average 31.1% of variance (Table 4). Shannon diversity index was positively related to monthly NDVI at 1000 m. (F = 2.083, df = 1,433, p = 0.04) (Fig 7). Total mosquito abundance was positively related to inundation area at 2000 m. (F = 1.991, df = 1,433, p = 0.05) (Fig 7).

**Table 4:** Results of the Generalized Linear Model (binomial) of monthly mosquito presence for seven mosquito species vs Inundation surface and NDVI estimated at five buffers of different size.

Dependent variable	Independent variable	Estimate	Std.error	Z value	p	Explained deviance
***Culex perexiguus***	Intercept	0.18763	0.23157	0.810		21.7%
	Inundation area (500 m.)	-0.22123	0.04381	-5.050	<0.0001	
	NDVI (500 m.)	7.63240	1.51660	5.033	<0.0001	
***Culex pipiens***	Intercept	-0.9572	0.1603	-5.970	<0.0001	21.1%
	NDVI (2000 m.)	6.6381	1.5025	4.418	<0.0001	
***Culex modestus***	Intercept	-2.8366	1.0358	-2.739	0.00617	14.4%
	Inundation area month before (2000 m.)	0.3981	0.1588	2.507	0.01217	
	NDVI month before (2000 m.)	-5.0727	1.4096	-3.599	0.00032	
***Culex theileri***	Intercept	-4.4830	0.9582	-4.678	<0.0001	49.7%
	Inundation area month before (2000 m.)	0.8981	0.1491	6.022	<0.0001	
	NDVI (2000 m.)	6.9586	2.0493	3.396	0.000685	
***Anopheles atroparvus***	Intercept	-2.4113	0.7257	-3.323	0.000891	43.6%
	Inundation surface (2000 m.)	0.2298	0.1136	2.023	0.043065	
	NDVI month before (2000 m.)	12.2210	1.6075	7.603	<0.0001	
***Ochlerotatus caspius***	Intercept	5.2698	0.8833	5.966	<0.0001	30.5%
	NDVI (2000 m.)	-12.1802	1.7969	-6.778	<0.0001	
	Inundation area (2000 m.)	-0.5996	0.1350	-4.441	<0.0001	
***Ochlerotatus detritus***	Intercept	-4.9594	0.9768	-5.077	<0.0001	36.7%

## Discussion

In order to grasp the complexities of mosquito-borne disease risk we need to understand the environmental factors underlying the distribution, abundance and composition of mosquito vector species that ultimately determine the distribution of vector-borne pathogens and make an important contribution to explaining the risk of pathogen transmission [39]. Given that measuring mosquito abundances in the field is labour-intensive and expensive [40], remote sensing data (satellite images) offer a cost-effective source of environmental data that can be related to vector distribution and abundance, these being effective models for predicting mosquito abundance [41]. In this study we investigated the relationships between landscape variables derived from satellite images and mosquito presence, abundance and diversity in a Mediterranean wetland. For most of the species, these models explained a considerable amount of variance compared with other models [41], so we were able to conclude that remote sensing is a powerful tool for estimating landscape variables that are related to mosquito distribution in natural wetlands.

Hydroperiod (water permanence) was negatively related to the annual presence of mosquito species (*Cx*. *pipiens*, *Cx*. *perexiguus*, *An*. *atroparvus*, *Oc*. *caspius and Oc*. *detritus*) and to mosquito species richness. In fact, we found ephemeral ponds to be the wetland habitats with greatest mosquito presence and richness, in agreement with other studies [42]. However, this study is the first, to our knowledge, to demonstrate that low hydroperiod measured from Landsat images can be a proxy for mosquito presence and richness. In addition, hydroperiod was positively related to the annual mosquito abundance of *Cx*. *theileri*, *Cx*. *pipiens* and *Cx*. *modestus*, the latter species identified in areas with long hydroperiods (over 150–200 days/year). Inundation area (water surface) was positively related to monthly mosquito presence and mosquito richness, except for the two salt marsh mosquitoes (*Oc*. *caspius* and *Oc*. *detritus*).

Both estimators, flooded area (inundation area) and flood duration (hydroperiod) determine desiccation and predation, and therefore affect mosquito communities in temporary ponds. To complete their larval aquatic cycle, mosquitoes must emerge before the pond dries. Ephemeral ponds (with low hydroperiods) have a high risk of desiccation but a lower probability of having mosquito predators, such as fish and aquatic insects (Heteroptera, Odonata, Coleoptera) [42]. Mosquitoes may be able to assess the risk of desiccation from inundation surface enabling them to evaluate the risk of predation and avoid ovipositing in permanent ponds (high hydroperiods) which have more abundant and diverse predators [43, 44]. Mosquito abundance may be high in areas where pools last long enough for mosquitoes to develop and emerge as adults, but not long enough for predators to colonize those habitats. In these semi-permanent ponds desiccation can produce pond fragmentation, optimal habitats for mosquitoes with low presence of predators and flooding lasting long enough for mosquitoes to complete their life cycle.

We detected species-specific differences in these patterns of habitat requirement. The high abundance threshold of *Cx*. *modestus* corresponded to hydroperiods over 150–200 days that were associated to a mosquito bloom in permanent marshlands in June. Two mosquito species were negatively related to inundation surfaces because they depend on water supply from tides (*Oc*. *caspius*) or prefer highly ephemeral ponds (*Cx*. *perexiguus*). In Doñana, there has been a trend to shorter annual hydroperiods in recent years due to aquifer over exploitation [34]. This may have complex effects on mosquito spatial distribution, and may increase the number of ephemeral ponds and the presence and richness of mosquitoes. We feel it very important to point out that in other areas where anthropic influence is greater than in these natural wetlands, the emergence of mosquito species may not (in general) match hydroperiod or rainfall patterns because their habitats include bodies of water flooded by human activity, such as peridomestic containers or agricultural channels.

NDVI is a landscape index of photosynthetic activity, primary productivity [12, 15, 45] and vegetation cover. Vegetation is important as resting habitat for both for adult mosquitoes and for their host vertebrates (such as birds). NDVI had a positive relationship with annual and monthly mosquito diversity (Shannon diversity Index), annual presence (*Cx*. *pipiens* and *An*. *atroparvus*), annual abundance (*Cx*.*perexiguus* and *Cx*. *pipiens*) and monthly presence (*Cx*. *perexiguus*, *Cx*. *pipiens*, *Cx*. *theileri* and *An*. *atroparvus*). However, NDVI was negatively related to the annual and monthly presence and abundance of the two saltmarsh species (*Oc*. *caspius* and *Oc*. *detritus*) because their optimal larval habitats are halophytic vegetation with *Spartina* and *Salicornia*, characterised by low NDVI values. In addition, *Cx*. *modestus* is again an exception, due to the mosquito bloom in permanent marshlands in June. As this species is related to permanent water, the presence and abundance of *Cx*. *modestus* was related to NDVI values over 0.2 and high inundation surfaces. These seasonal mosquito patterns are related to climatic factors [36].

This study also sought to determine whether the monitoring of landscape variables to predict the risk of mosquito abundance, presence and diversity was best done at a micro-scale or a meso-scale, and this emerges from the buffer analysis. The best model was in general the 2000 metre buffer, this being the scale that gives most landscape information. The optimal scale is therefore not necessarily related to the flight range of the mosquito species or the fine resolution of the data, but rather to characteristics of the landscape variables. A large scale provides better models probably because better reflect relevant patterns of landscape variation important for mosquito reproduction and feeding (i.e. related to host, vegetation, predators or desiccation patterns). The pattern that large scales are more predictive patterns has already been detected by other authors [17, 35, 46]. The practical implications of these results are that the scale of the analysis should not be so fine as to under-represent the spatial variability of the factors driving mosquito distribution and abundance.

The influence of landscape variables on disease transmission risk for mosquito-borne pathogens has not been fully explicated, but there is clearly a link between landscape, mosquito populations, and mosquito-borne disease occurrence [5]. It has been suggested that greater wetland area and increasing hydrogeographic area are associated with decreasing WNV cases [19,47], and that drought conditions favour the presence of WNV in mosquitoes [48,49]. This drought-WNV relationship may be due to drought influencing the concentrations of birds and mosquitoes near water sources or affecting the vector competence of mosquitoes. Our results provide an alternative explanation, given the relation we found between high mosquito presence and richness and areas with short hydroperiods. However, these relationships are complex, as revealed by our finding that high inundation surfaces condition high mosquito abundances of most *Culex* species when monthly data are considered, indicating that the differences are not only spatially but also temporally conditioned and clearly depend on the ecology of each vector species.

Our results show that higher NDVI values may condition higher *Culex* presences and abundances, which may in turn influence WNV disease risk. Our data show that the abundance-NDVI relationship is species-specific and cannot be generalised, and therefore priority must be given to specific vector species according to their vector competence. In the USA, urban land cover and WNV have been found to be related [17], and Bowden et al (2011) [9] detected different patterns in north-eastern and western USA. They concluded that the effect of landscape on human disease risk is primarily mediated by its effect on the vector community and the different mosquito species vectors. This landscape-mosquito community effect may be mediated by complex interactions between hydroperiod, inundation surface and NDVI across different Landscape Units. This effect of landscape on mosquito abundance must be taken into account when planning conservation management in Doñana and other important wetlands. Landscape changes (for example, increasing halophytic marshes) likely have significant effects on mosquito communities. An important result of this study is that different species of mosquitoes have completely different habitat requirements resulting in completely different distributions. As can be observed in Fig 2, areas with higher abundances of each mosquito species seldom overlap. The practical implication is clear: taking these habitat preferences into account when designing mosquito control strategies in order to reduce the risk of transmission of specific diseases may greatly increase the efficacy of management actions while reducing economic and environmental costs. Consequently, when dealing with disease outbreaks, the objectives should be not only to control the number of mosquitoes but identify the mosquito species target and to reduce in particular populations of mosquito species that play a greater role in amplification and transmission of the pathogen in question to humans or other species of interest.

To sum up, we found strong evidence that mosquito vector abundance, presence and diversity are related to landscape variables. Water and vegetation distribution across the landscape may be of particular importance in the distribution of mosquitoes and the interaction with host distribution. We detected some common patterns among mosquito species, but each has its own bionomic properties (flight range, interspecific larval competition, larval habitat preferences, host preferences, etc.) conditioning specific relationships with landscape indicators. We found evidence that, usually, larger buffers captured better environmental variability related to mosquito abundance and distribution patterns.

This extensive study involved the capture of large numbers of mosquitoes in more than one hundred sites. However, further studies are needed to improve on certain limitations in this work, such as including survey sin urban and periurban landscapes, extending the sampling period over several years or developing extensive larval surveys. Only global approaches can help us understand the complex inter-relationships between pathogens, vectors, hosts and environment that determine the transmission cycles of mosquito-borne diseases. In particular, understanding the link between landscape and vector populations is central in the design of mosquito control measures to prevent disease outbreaks, and in understanding where and when the risk of mosquito-borne disease is highest.

## Supporting Information

S1 Data Annual database table. (XLSX)

S2 Data Monthly database table. (XLSX)

## References

[1] Rezza G, Nicoletti L, Angelini R, Romi R, Finarelli AC, Panning M, et al Infection with chikungunya virus in Italy: an outbreak in a temperate region. Lancet 2007; 370: 1840–1846. 18061059 10.1016/S0140-6736(07)61779-6

[2] Gould EA, Higgs S. Impact of climate change and other factors on emerging arbovirus diseases. Trans R Soc Trop Med Hyg. 2009; 103: 109–121. 10.1016/j.trstmh.2008.07.025 18799177 PMC2915563

[3] Danis K, Papa A, Papanikolaou E, Dougas G, Terzaki I, Baka A, et al Ongoing outbreak of West Nile virus infection in humans, Greece, July to August 2011. Euro Surveill. 2011; 16(34): 2752–2762. 21903037

[4] Vazquez A, Jimenez-Clavero M, Franco L, Donoso-Mantke O, Sambri V, Niedrig M, et al Usutu virus—potential risk of human disease in Europe. Euro Surveill. 2011; 16 (31): 1–5. 21871214

[5] Ostfeld RS, Glass GE, Keesing F. Spatial epidemiology: an emerging (or re-emerging) discipline. Trends Ecol Evol. 2005; 20: 328–336. 16701389 10.1016/j.tree.2005.03.009

[6] Kilpatrick AM. Globalization, land use, and the invasion of West Nile virus. Science. 2011; 334: 323–327. 10.1126/science.1201010 22021850 PMC3346291

[7] Muñoz J, Ruiz S, Soriguer R, Alcaide M, Viana DS, Roiz D, et al Feeding Patterns of Potential West Nile Virus Vectors in South-West Spain. PLoS One. 2012; 7(6): e39549 10.1371/journal.pone.0039549 22745781 PMC3382169

[8] Roche B, Rohani P, Dobson AP, Guégan J-F. The Impact of Community Organization on Vector-Borne Pathogens. Am Nat. 2013; 181: 1–11. 10.1086/668591 23234841

[9] Kilpatrick AM, Kramer LD, Jones MJ, Marra PP, Daszak P. West Nile virus epidemics in North America are driven by shifts in mosquito feeding behaviour. PLoS Biol. 2006; 4: 606–610. 10.1371/journal.pbio.0040082 PMC1382011 16494532

[10] Simpson JE, Hurtado PJ, Medlock J, Molaei G, Andreadis TG, Galvani AP, et al Vector host-feeding preferences drive transmission of multi-host pathogens: West Nile virus as a model system. Proc R Soc Lond. 2012; 279: 925–933. 10.1098/rspb.2011.1282 21849315 PMC3259921

[11] Smith DL, Dushoff J, McKenzie FE. The Risk of a Mosquito-Borne Infection in a Heterogeneous Environment. PLoS Biol. 2004; 2(11): e368 15510228 10.1371/journal.pbio.0020368 PMC524252

[12] Kitron U.Landscape ecology and epidemiology of vector-borne disease: tools for spatial analysis. J Med Entomol. 1998; 35: 435–445. 9701925 10.1093/jmedent/35.4.435

[13] Winters AM, Eisen RJ, Lozano-Fuentes S, Moore CG, Pape WJ, Eisen L.l. Predictive Spatial Models for Risk of West Nile Virus Exposure in Eastern and Western Colorado. Am J Trop Med Hyg. 2008; 79: 581–590. 18840749 PMC2581834

[14] Crowder DW, Dykstra EA, Brauner JM, Duffy A, Reed C, Martin E, et al West Nile virus prevalence across landscapes is mediated by local effects of agriculture on vector and host communities. PloS One, 2013; 8(1), e55006 10.1371/journal.pone.0055006 23383032 PMC3559328

[15] Brownstein JS, Rosen H, Purdy D, Miller JR, Merlino M, Mostashari F, et al Spatial Analysis of West Nile Virus: Rapid Risk Assessment of an Introduced Vector-Borne Zoonosis. Vector Borne Zoonotic Dis. 2002; 2: 157–164. 12737545 10.1089/15303660260613729

[16] Diuk-Wasser MA, Brown HE, Andreadis TG, Fish D. Modeling the Spatial Distribution of Mosquito Vectors for West Nile Virus in Connecticut, USA. Vector Borne Zoonotic Dis. 2006; 6: 283–295. 16989568 10.1089/vbz.2006.6.283

[17] Brown H, Diuk-Wasser M, Andreadis T, Fish D. Remotely-Sensed Vegetation Indices Identify Mosquito Clusters of West Nile Virus Vectors in an Urban Landscape in the Northeastern United States. Vector Borne Zoonotic Dis. 2008; 8: 197–206. 10.1089/vbz.2007.0154 18452400

[18] Bustamante J, Pacios F, Díaz-Delgado R, Aragonés D. Predictive models of turbidity and water depth in the Doñana marshes using Landsat TM and ETM+ images. J Environ Manage. 2009; 90: 2219–2225. 10.1016/j.jenvman.2007.08.021 18395320

[19] Cleckner H. L., Allen T. R., & Bellows A. S. Remote sensing and modeling of mosquito abundance and habitats in Coastal Virginia, USA. Remote Sensi. 2011; 3(12), 2663–2681.

[20] BØgh C., Lindsay S. W., Clarke S. E., Dean A., Jawara M., Pinder M., et al High spatial resolution mapping of malaria transmission risk in the Gambia, west Africa, using LANDSAT TM satellite imagery. Am J Trop Med Hyg. 2007; 76(5), 875–881. 17488908

[21] Ezenwa VO, Milheim LE, Coffey MF, Godsey MS, King RJ, Guptill SC, et al Land Cover Variation and West Nile Virus Prevalence: Patterns, Processes, and Implications for Disease Control. Vector Borne Zoonotic Dis. 2007; 7: 173–180. 17627435 10.1089/vbz.2006.0584

[22] Figuerola J, Jimenez-Clavero MA, Lopez G, Rubio C, Soriguer R, Gómez-Tejedor C, et al Size matters: West Nile Virus neutralizing antibodies in resident and migratory birds in Spain. Vet Microbiol. 2008; 132: 39–46. 10.1016/j.vetmic.2008.04.023 18514437

[23] Figuerola J, Angel Jiménez-Clavero M, Rojo G, Gómez-Tejedor C, Soriguer R. Prevalence of West Nile virus neutralizing antibodies in colonial aquatic birds in southern Spain. Avian Pathol. 2007; 36: 209–212. 17497333 10.1080/03079450701332329

[24] Jiménez-Clavero MA, Concepción Gómez T, Rojo G, Soriguer R, Figuerola J (2007) Serosurvey of West Nile virus in equids and bovids in Spain. Vet Rec. 2007; 161: 212 17693638 10.1136/vr.161.6.212

[25] Garcia-Bocanegra I, Jaen-Tellez JA, Napp S, Arenas-Montes A, Fernandez-Morente M, Fernández-Molera V, et al West Nile Fever Outbreak in Horses and Humans, Spain, 2010. Emerg Infect Dis. 2011; 17: 2397–2399. 10.3201/eid1712.110651 22172565 PMC3311180

[26] Vázquez A, Sánchez-Seco MP, Ruiz S, Molero F, Hernández L, Moreno J, et al Putative New Lineage of West Nile Virus, Spain. Emerg Infect Dis. 2010; 16 (3): 549–552. 10.3201/eid1603.091033 20202444 PMC3322021

[27] Vazquez A, Ruiz S, Herrero L, Moreno J, Molero F, Magallanes A, et al West Nile and Usutu viruses in mosquitoes in Spain, 2008–2009. Am J Trop Med Hyg. 2011; 85: 178–181. 10.4269/ajtmh.2011.11-0042 21734145 PMC3122364

[28] Vázquez A, Sánchez-Seco M-P, Palacios G, Molero F, Reyes N, Ruiz S, et al Novel Flaviviruses Detected in Different Species of Mosquitoes in Spain. Vector Borne Zoonotic Dis. 2011; 12(3): 223–229. 10.1089/vbz.2011.0687 22022811 PMC3300060

[29] Reisen WK, Lothrop HD. Effects of sampling design on the estimation of adult mosquito abundance. J Am Mosq Control Assoc. 1999; 15: 105–114. 10412106

[30] Roiz D, Roussel M, Muñoz J, Ruiz S, Soriguer R, Figuerola J.. Efficacy of Mosquito Traps for Collecting Potential West Nile Mosquito Vectors in a Natural Mediterranean Wetland. Am J Trop Med Hyg.2012; 86: 642–648. 10.4269/ajtmh.2012.11-0326 22492149 PMC3403756

[31] Schaffner F, Angel G, Geoffroy B, Hervy JP, Rhaiem A, Brunhes J. et al Les moustiques d’Europe/The mosquitoes of Europe. CD-ROM 2011 Montpellier, France: Institut de Recherche pour le Développement/EID Méditerranée.

[32] Becker N, Petric D, Zgomba M, Boase C, Madon M, Dahl C. et al Mosquitoes and Their Control. Springer Science & Business Media; 2010.

[33] Harbach R. E. The identity of *Culex perexiguus* Theobald versus ex. univittatus Theobald in southern Europe. Eur Mosq Bull. 1999; 4(7).

[34] Gómez-Rodríguez C, Bustamante J, Díaz-Paniagua C. Evidence of Hydroperiod Shortening in a Preserved System of Temporary Ponds. Remote Sens. 2010; 2: 1439–1462.

[35] McAlpine CA, Rhodes JR, Bowen ME, Lunney D, Callaghan JG, Mitchell DL, et al Can multiscale models of species’ distribution be generalized from region to region? A case study of the koala. J App Ecol. 2008; 45(2), 558–567.

[36] Roiz D, Ruiz S, Soriguer R, Figuerola J. Climatic effects on mosquito abundance in Mediterranean wetlands. Parasit Vectors. 2014; 7(1): 333. 25030527 10.1186/1756-3305-7-333 PMC4223583

[37] Crawley MJ. Statistical computing: an introduction to data analysis using S-Plus: John Wiley and Sons; 2002.

[38] Zuur AF, Ieno EN, Elphick CS. A protocol for data exploration to avoid common statistical problems. Methods Ecol Evol. 2010; 1: 3–14.

[39] Lambin EF, Tran A, Vanwambeke SO, Linard C, Soti V. Pathogenic landscapes: interactions between land, people, disease vectors, and their animal hosts. Int J Health Geogr. 2010; 9(54), 1–13. 20979609 10.1186/1476-072X-9-54 PMC2984574

[40] Hay SI, Packer MJ, Rogers DJ. The impact of remote sensing on the study and control of invertebrate intermediate hosts and vectors for disease. Int JRemote Sens. 1997; 18(14), 2899–2930.

[41] Reisen WK. Landscape epidemiology of vector-borne diseases. Ann Rev Entomol. 2010; 55, 461–483. 10.1146/annurev-ento-112408-085419 19737082

[42] Arav D, Blaustein L. Effects of Pool Depth and Risk of Predation on Oviposition Habitat Selection by Temporary Pool Dipterans. J Med Entomol. 2006; 43: 493–497. 16739406 10.1603/0022-2585(2006)43[493:eopdar]2.0.co;2

[43] Silberbush A, Blaustein L. Mosquito females quantify risk of predation to their progeny when selecting an oviposition site. Func Ecol. 2011; 25: 1091–1095.

[44] Corti D, Kohler SL, Sparks RE. Effects of hydroperiod and predation on a Mississippi River floodplain invertebrate community. Oecologia. 1996; 109: 154–165. 28307606 10.1007/s004420050070

[45] Pettorelli N, Ryan S, Mueller T, Bunnefeld N, Jdrzejewska B, Lima M, et al The Normalized Difference Vegetation Index (NDVI): unforeseen successes in animal ecology. Clim Res. 2011; 46: 15–27.

[46] Schafer ML, Lundstrom JO, Pfeffer M, Lundkvist E, Landin J. Biological diversity versus risk for mosquito nuisance and disease transmission in constructed wetlands in southern Sweden. Med Vet Entomol. 2004; 18: 256–267. 15347393 10.1111/j.0269-283X.2004.00504.x

[47] Walsh MG. The Role of Hydrogeography and Climate in the Landscape Epidemiology of West Nile Virus in New York State from 2000 to 2010. PLoS One. 2012; 7: e30620 10.1371/journal.pone.0030620 22328919 PMC3273478

[48] Shaman J, Day JF, Stieglitz M. Drought-Induced Amplification and Epidemic Transmission of West Nile Virus in Southern Florida. J Med Entomol. 2005; 42: 134–141. 15799522 10.1093/jmedent/42.2.134

[49] Bowden S, Magori K, Drake J. Regional differences in the association between Land Cover and West Nile Virus Disease Incidence in Humans in the United States. Am J Trop Med Hyg. 2011; 84(2), 234–238 10.4269/ajtmh.2011.10-0134 21292890 PMC3029173

